# Impact of a Residential Summer Camp Experience on Children With Sickle Cell Disease

**DOI:** 10.31486/toj.20.0149

**Published:** 2021

**Authors:** Adam Paul Yan, Monakshi Sawhney, Melanie Kirby-Allen

**Affiliations:** ^1^ Division of Hematology/Oncology, The University of Toronto, Toronto, Ontario, Canada; ^2^ The Hospital for Sick Children, Toronto, Ontario, Canada; ^3^ Division of Anesthesia and Perioperative Medicine, Queens University, Kingston, Ontario, Canada

**Keywords:** *Anemia–sickle cell*, *child*, *healthcare disparities*, *residential camp*

## Abstract

**Background:** Sickle cell disease (SCD) has a significant psychosocial impact on affected children. Summer camp has been shown to improve psychological and physical states for children with diabetes and cancer. However, opportunities to attend camp for children with SCD are limited, as many are from low-income families, and many camps are not equipped to care for children with medical complexities. To our knowledge, no literature evaluates how camp can positively affect emotional functioning, social functioning, self-esteem, and physical activity levels in children with SCD.

**Methods:** Children with SCD attending a residential summer camp during 2019 were identified. Participants completed a modified version of the Pediatric Camp Outcome Measure, a validated 29-item questionnaire that evaluates self-esteem, emotional function, social function, and physical activity. Four additional questions related to SCD were included.

**Results:** Nine campers enrolled in the study. Questionnaire results showed a total score of 113.7 (maximum score135, range 84-129), with a self-esteem subscale score of 22.1 (maximum score 25, range 20-25), an emotional subscale score of 32.1 (maximum score 40, range 25-39), a social subscale score of 38.9 (maximum score 45, range 24-45), and a physical activity subscale score of 20.6 (maximum score 25, range 19-25). All campers indicated that they would return to camp.

**Conclusion:** Attending summer camp has a beneficial impact on emotional function, social function, self-esteem, and physical activity. Mean questionnaire scores from children with cardiac disease and cancer are similar to those of children with SCD. Increased funding should be awarded to sickle cell camps to allow for more children to have this beneficial experience.

## INTRODUCTION

Sickle cell disease (SCD) is one of the most common, severe, monogenic disorders affecting children in Canada.^1^ SCD affects 1 in every 500 infants of African ancestry.^2^ In SCD, a point mutation in the beta-globin gene causes the production of abnormal sickled hemoglobin. Affected individuals have numerous medical complications, including severe infections, acute and chronic pain, acute chest syndromes, strokes, retinopathy, avascular necrosis, and nephropathy.^3^

Having a complex chronic illness has a significant psychological impact on children with SCD. Children with SCD have decreased school attendance, decreased participation in school and social activities, and poor adaptation compared to peers without SCD.^4-8^ Chronically ill children may also suffer social deprivation as a result of prolonged hospitalizations. Many children with SCD have low self-esteem and are worried about looking different than their peers.^9^ Caring for a child with SCD can also cause significant strain on family members. Caregivers of children with SCD report more fatigue, more difficulty concentrating, and struggle more with stress compared to the caregivers of children with other chronic and complex health conditions.^10^

To help mitigate the psychosocial impact of SCD on a child, providing opportunities to children with SCD to develop their self-confidence and resiliency skills is important. Children with cancer who participated in a residential camping experience showed improvement in social acceptance, physical appearance, and global self-worth.^11^ In children with other chronic diseases, residential summer camp has been shown to decrease anxiety, increase self-esteem, improve quality of life, and improve physical symptoms.^12-14^ Camp also provides children with chronic diseases an opportunity to practice managing their illness and meet children who share their diagnosis. Among children with type 1 diabetes, attending summer camp increased self‐efficacy and resilience, improved knowledge, and improved diabetes self‐management skills, leading to improved glycemic control.^15^ The positive effects of residential camp are not transient and may be retained weeks to months after leaving camp.^16,17^

In a 2018 study, pediatric health care professionals uniformly felt that residential camps for children and teens with SCD and cancer had a positive influence on campers’ normalization and health care ownership and helped strengthen bonds between patients and providers.^18^ Camp also offers an opportunity for care coordination, dissemination of best practices for the disease, education on self-care, and help with the transition from adolescent to adult care.^19^

However, the opportunity for children with SCD to attend summer camp is limited. In the United States, 67% of children with SCD live in poverty or low-income families,^20^ and the same is believed to be true for children with SCD living in Canada; however, no data are available that characterize the socioeconomic status of patients with SCD in Canada. Because of these financial restraints, many families cannot afford the luxury of summer camp. Even for families that can afford camp, the options may be limited, as many camps may not be equipped to care for medically complicated children. In response to these barriers, numerous camps have emerged with the mandate to provide a free camping experience for children with SCD.

Camp Jumoke is a fundraising organization in Ontario, Canada, with a mission to raise money to send children with SCD to summer camp at Camp Wenonah, the only residential summer camp program for children with SCD in Canada. Camp Jumoke covers the complete cost of summer camp (approximately $2,300 per child) and organizes for trained medical professionals to be onsite. The SCD campers are embedded within a larger residential summer camp that is primarily attended by children who do not have an SCD diagnosis or any other chronic medical illness. The SCD campers are integrated into cabins with some children who have SCD and others who do not. Consequently, throughout their stay, campers with SCD sleep, eat, and participate in camp activities with campers who do not have SCD.

Little research has been published regarding the impact of camp on children and families with SCD. To our knowledge, only 3 studies have examined outcomes of residential camps for children with SCD. The first was a pilot project conducted in 1979 to assess the possibility of SCD children attending camp. The study concluded that children with SCD could attend a regular camp and participate in most activities.^21^ The second study was a 22-year review of medically related concerns of a camp that provided a camping experience to more than 1,000 children with SCD.^22^ The study found that 10% of campers had SCD-related medical problems at camp, and 3% of campers required transfer to a hospital for SCD management while at camp.^22^ The third study utilized a post-camp survey to assess the impact of an H1N1 virus pandemic outbreak at a camp for children with cancer and hematologic conditions and their siblings. Morrison et al reported that of 165 campers and staff, 38.5% (n=59) reported flu-like symptoms, and 82% of children with SCD at the camp reported flu-like symptoms.^23^

These studies assessed the safety of residential camps for children with SCD but failed to evaluate the utility of camp in affecting the participants’ emotional function, social function, self-esteem, and physical activity levels. The aim of this study was to examine the physical and psychological impact of attending residential camp among children and teens attending Camp Wenonah in Ontario, Canada. We hypothesized that campers with SCD would report positive emotional function, social function, self-esteem, and physical activity levels.

## METHODS

This study was conducted in August 2019 at Camp Wenonah during the last 2 days of camp. Research ethics board approval (REB#1000063779) was obtained from The Hospital for Sick Children to conduct this study. Informed consent was obtained from the child's parent or guardian by telephone prior to the start of camp. Assent was obtained from the child at camp after the study aim, and procedures were explained and prior to completing the outcome measures.

### Participants

Children were eligible for inclusion if they had a diagnosis of hemoglobin SS, SC, Sβ^+^ thalassemia, or Sβ^0^ thalassemia SCD subtypes; were 8 to 16 years of age at the time of attending camp; and attended Camp Wenonah for at least 1 week during summer 2019.

### Outcome Measures

Outcomes were measured using the Pediatric Camp Outcome Measure (PCOM), a 29-item, self-report questionnaire assessing children's camp experiences. The PCOM was initially developed in 2008 at Stanford University. The total scale has shown strong internal consistency (Cronbach alpha=0.93), as has each of the subscales (alpha ranging from 0.39 to 0.79). Following initial item selection, analysis of variance and Pearson product-moment correlation coefficients showed no differences in PCOM score between age, sex, and disease severity. Correlation analysis between PCOM and established measures of depression (Children's Depression Inventory), anxiety (Revised Children's Manifest Anxiety Scale), and generic quality of life (Pediatric Quality of Life Inventory) showed that PCOM scores correlated with these established scales.^24^ The PCOM was validated in a second group of camps and again showed high internal consistency and construct validity.^24,25^

Twenty-seven items contribute to 4 subscales: (1) emotional function, (2) social function, (3) self-esteem, and (4) physical activity. These 27 items contribute to a total score and are answered using a 5-point Likert-type scale (1=almost never or very bad/sad/hard; 5=almost always or very good/happy/easy). The PCOM also includes 2 additional questions that ask participants what they would tell other children about camp and if they would return to camp. These 2 questions are not included in the PCOM scores. For this study, 4 additional questions that specifically assessed how having SCD affected the campers’ experiences were added to the end of the PCOM. Two of these questions were yes or no response questions; the other 2 used the same Likert-type scale as the PCOM for responses.

### Statistical Analysis

Demographic data and clinical characteristics were analyzed using descriptive statistics, reporting means, ranges, and proportions. The responses to each item on the questionnaire were aggregated and are presented as means with ranges as appropriate. Results were compared to PCOM scores from prior studies conducted at camps for children with cancer and congenital heart disease.^24,25^

## RESULTS

### Cohort Characteristics

Ten individuals were identified as eligible for this study, and all 10 consented to participate. Nine participants completed the final questionnaire. One participant left camp unexpectedly and was therefore unable to complete the questionnaire. The mean age of participants was 12.7 years. The majority of participants were male (66.7%, n=6), had a sickle cell diagnosis of hemoglobin SS (55.6%, n=5), and had attended Camp Wenonah before (66.7%, n=6). Demographic data for participants are displayed in Table 1.

**Table 1.** Demographic Data, n=9

Characteristic	n (%)	Mean
Sex		
Male	6 (66.7)	
Female	3 (33.3)	
Sickle cell genotype		
Hemoglobin SS	5 (55.6)	
Hemoglobin SC	4 (44.4)	
Hemoglobin Sβ^+^ thalassemia	0	
Hemoglobin Sβ^0^ thalassemia	0	
Age, years		12.7
8-12	2 (22.2)	
13-16	7 (77.8)	
Prior Camp Jumoke attendance		
Yes	6 (66.7)	
No	3 (33.3)	
Number of times attending Camp Jumoke^a^		4.5
Prior attendance at other camps		
Yes	2 (22.2)	
No	7 (77.8)	

^a^Only calculated for campers who had been to camp before (n=6).

### Pediatric Camp Outcome Measure Scores

The mean total PCOM score was 113.7 (maximum score 135, range 84-129). The mean PCOM subscale scores were 22.1 (maximum score 25, range 20-25) for self-esteem, 32.1 (maximum score 40, range 25-39) for emotional function, 38.9 (maximum score 45, range 24-45) for social function, and 20.6 (maximum score 25, range 19-25) for physical activity. PCOM scores of campers with SCD are compared to PCOM scores for children with congenital heart disease and cancer in Table 2, and full questionnaire results for this study are displayed in Table 3.

**Table 2.** Comparison of Mean Pediatric Camp Outcome Measure Scores Between Campers With Sickle Cell Disease, Congenital Heart Disease, and Cancer

Subscale and Total	Sickle Cell Disease (n=9)	Congenital Heart Disease^24^ (n=51)	Cancer^25^ (n=1,230)	Possible Range
Self-esteem	22.1	20.4	22.5	5-25
Emotional	32.1	32.9	35.0	8-40
Social	38.9	36.4	39.9	9-45
Physical	20.6	18.0	20.5	5-25
Total score	113.7	107.7	117.9	27-135

Notes: Mean scores are presented. Higher numbers represent a more positive response.

**Table 3.** Pediatric Camp Outcome Measure Questionnaire Results, n=9

	Number of Responses					
	by Likert Scale					Mean
Question	1	2	3	4	5	Score
**Self-esteem subscale**						
How often did you feel like yourself at camp? (1=almost never; 5=almost always)	0	0	0	4	5	4.5
How did you feel about yourself at camp? (1=very bad; 5=very good)	0	0	2	4	3	4.1
How often were you proud of yourself at camp? (1=almost never; 5=almost always)	0	0	1	3	5	4.4
How often did you like yourself at camp? (1=almost never; 5=almost always)	0	0	0	2	7	4.8
How often did you feel like you could do the activities the other kids at camp were doing? (1=almost never; 5=almost always)	0	0	1	5	3	4.2
**Emotional functioning subscale**						
How happy or sad were you at camp? (1=very sad; 5=very happy)	0	0	1	4	4	4.3
How often were you nervous at camp? (1=almost always; 5=almost never)^a^	0	0	3	1	5	4.2
How often did you worry at camp? (1=almost always; 5=almost never)^a^	0	0	4	3	2	3.8
How often did you worry about your health condition at camp? (1=almost always; 5=almost never)^a^	1	1	2	4	1	3.3
How often did you worry about what the other kids at camp thought about you? (1=almost always; 5=almost never)^a^	1	2	1	2	3	3.4
How often did you feel sad or blue at camp? (1=almost always; 5=almost never)^a^	0	0	1	3	5	4.4
How often did you feel homesick at camp? (1=almost always; 5=almost never)^a^	0	1	4	0	4	3.8
How much did you like or dislike camp? (1=I hated it; 5=I really liked it)	0	0	1	0	8	4.8
**Social functioning subscale**						
How often were you lonely at camp? (1=almost always; 5=almost never)^a^	0	2	1	1	5	4.0
How often did you spend time with your friends at camp? (1=almost never; 5=almost always)	0	0	1	2	6	4.6
How often did you have someone to talk to at camp? (1=almost never; 5=almost always)	0	0	1	0	8	4.8
What was it like to make friends at camp? (1=very hard; 5=very easy)	1	0	1	1	6	4.2
What was it like to play with kids you did not know very well? (1=very hard; 5=very easy)	1	0	2	2	4	3.9
How often did you play with the other kids at camp? (1=almost never; 5=almost always)	0	0	2	0	7	4.6
How often did you feel like you were part of the group at camp? (1=almost never; 5=almost always)	0	0	1	2	6	4.6
How often did you feel left out at camp? (1=almost never; 5=almost always)^a^	0	1	2	2	4	4.0
How often did you get along with the other kids at camp? (1=almost never; 5=almost always)	0	1	0	3	5	4.3
**Physical functioning subscale**						
How often were you active at camp? (1=almost never; 5=almost always)	0	0	1	2	6	4.6
How often did you feel like you had energy at camp? (1=almost never; 5=almost always)	0	0	0	4	5	4.6
How often did you do sports activities at camp? (1=almost never; 5=almost always)	0	2	0	2	5	4.1
How often did you exercise at camp? (1=almost never; 5=almost always)	0	1	1	1	6	4.3
How often did you get tired and have to sit down at camp? (1=almost always; 5=almost never)^a^	0	3	3	2	1	3.1
**Additional questions**						
What would you tell other kids about camp? (1=it was very bad; 5=it was very good)	0	0	1	0	8	4.8
Do you feel there were any activities you could not do because of your sickle cell disease? (1=almost always; 5=almost never)^a^	0	1	1	4	3	4.0
Did having sickle cell disease make you feel different than the other kids at camp? (1=almost always; 5=almost never)^a^	0	1	1	1	6	4.3
Would you want to come back to camp next year? (Yes; No)^b^	Yes=8, No=0					N/A
Did you learn anything new about sickle cell while at camp? (Yes; No)	Yes=5, No=4					N/A
Did you learn anything new about yourself while at camp? (Yes; No)	Yes=9, No=0					N/A

^a^Questions are negatively worded and scored in reverse.

^b^The n for this question equals 8 as 1 participant did not provide a response.

### Additional Questionnaire Results

The results of the 6 additional questions that do not contribute to the PCOM-scaled scores but were included in this study are summarized in Table 3. All participants indicated that they would come back to camp again and that they learned something new about themselves while at camp. The majority (56%) of participants indicated that they learned something new about SCD while at camp.

## DISCUSSION

To our knowledge, this study is the first to use a standardized measure to evaluate the impact of residential camping experiences for children with SCD. The mean PCOM score in our study was 113.7, which is similar to published PCOM scores from camps for children with congenital heart disease (107.7)^24^ and cancer (117.9).^25^ The similar PCOM scores indicate that attending camp is as beneficial for children with SCD as it is for children with other chronic medical conditions. Campers also felt that camp was a beneficial experience, with all participants indicating that they would like to return to camp.

Despite the clear benefits of camp, very few children with SCD can attend. Only 22% of campers had been to an alternative camp prior to attending Camp Wenonah. Children with SCD have more health economic inequalities compared to children with other chronic diseases. For instance, cystic fibrosis affects fewer than half the number of people that SCD does, but related organizations receive 440 times more funding.^26^ Funding that is specifically earmarked for children with chronic illness to attend residential camp also differs. Funding to send children with cancer to camp is significantly higher than funding to send children with SCD to camp.^27,28^

Further research on the effects of camp on children with SCD is necessary to create evidence-based rationale for increased community support and camp funding. Research should focus on evaluating which aspects of camp are beneficial for campers with SCD to further integrate similar experiences into the camp schedule. Given that 7 of 9 campers answered that they often or almost always felt that they could not do certain activities at camp because of their SCD, studies should assess what these activities are and evaluate modifications that can be made to allow children with SCD to participate. As noted earlier, this questionnaire was administered at a camp that was not uniquely tailored to children with SCD, and campers with SCD were integrated into cabins with children who did not have SCD. Seven of 9 campers answered that they often or almost always felt different than children without SCD at camp.

Whether having a camp exclusively dedicated to children with SCD would be more beneficial than having campers with SCD integrated into a generic summer camp is unknown. Being around other children who also have SCD may make affected youth feel less isolated and not feel like they are the only ones affected with chronic medical issues. While no formal educational sessions were provided to campers on the topic of SCD while at camp, 56% of campers indicated that they learned something new about SCD while at camp. At other disease-specific camps, attending camp has been shown to increase medication adherence and disease control.^15^ Further studies of SCD camps may seek to integrate formal learning experiences that focus on the importance of medication adherence to evaluate whether attending camp can alter medication compliance and disease control. Improved medication adherence would be particularly beneficial in the SCD population where compliance with disease-modifying drugs is often suboptimal.^29^

The principal limitation of our study is the small population size. Camp Wenonah had peak enrollment in 2015; 50 children with SCD attended camp that summer. Since 2015, enrollment has declined because of funding constraints, with only enough funding in 2019 to send 9 children to camp. In recent years, funding constraints have continued, limiting the number of children the camp can accommodate.

Despite the small number of participants in our study, however, all campers agreed to participate. As stated earlier, a child had to leave camp early because of a medical issue, resulting in 1 participant not completing the questionnaire. Another child handwrote an answer on the questionnaire instead of circling an answer, resulting in that question not being scored. We feel that patient recruitment was successful in our study because we focused on a topic that involved families are passionate about and because we minimized inconvenience to families by not requiring visits to the hospital for consent or completion of the questionnaire.

Another study limitation is that the 4 added questions specifically addressing how having SCD affected the camping experience have not been validated. In the future, we would like to perform a more robust study with more campers and additional health-related, quality-of-life, and self-efficacy measures.

## CONCLUSION

We determined that attending a residential summer camp has a positive effect on the emotional function, social function, self-esteem, and physical activity levels of children with SCD. The magnitude of the benefits seen is comparable to that demonstrated in studies of camps for children with other chronic diseases. However, in contrast to other disease-based camps, SCD camps receive a fraction of the funding and can only send a small number of children to camp. Our small sample size further highlights the dire need for individuals, hospitals, corporate entities, and government bodies to recognize the benefits of a summer camp experience and to fund summer camp experiences for children with SCD accordingly.

## Notes

*This article meets the Accreditation Council for Graduate Medical Education and the American Board of Medical Specialties Maintenance of Certification competencies for Patient Care, Medical Knowledge, and Practice-Based Learning and Improvement.*
